# Therapeutic potential of calcitriol in cerebral ischemia/reperfusion injury: In vivo and in silico insights into TLR4 and FGFR2 pathways

**DOI:** 10.1016/j.ibneur.2025.06.018

**Published:** 2025-07-29

**Authors:** Fahimeh Ramshini, Javad Amini Mahabadi, Reza Bayat, Sayyed Alireza Talaei, Zeinab Vahidinia, Hassan Hassani Bafrani

**Affiliations:** a Anatomical Sciences Research Center, Institute for Basic Sciences, Kashan University of Medical Sciences, Kashan, Iran; b Gametogenesis Research Center, Kashan University of Medical Sciences, Kashan, Iran; c Department of Cell and Molecular Biology, Faculty of Chemistry, University of Kashan, Kashan, Iran; d Physiology Research Center, Institute for Basic Sciences, Kashan University of Medical Sciences, Kashan, Iran

**Keywords:** Calcitriol, Ischemic stroke, Neuroinflammation, NF-kβ, TLR4, MyD88, FGFR2

## Abstract

**Background:**

Cerebral ischemic injury remains a major cause of high mortality, with limited effective treatments available. Inflammatory responses play a critical role in the pathophysiology of cerebral ischemia/reperfusion (I/R) injury. Suppressing inflammation is a key strategy for mitigating cerebral I/R injury, making it a promising therapeutic target for stroke. Vitamin D supplementation has been revealed to exhibit anti-inflammatory and neuroprotective properties during I/R injury; however, the underlying protective mechanisms are not yet fully understood. This study aimed to investigate the effects of post-ischemic calcitriol treatment on ischemic stroke, focusing specifically on the TLR4/MyD88/NF-κB and FGFR2 signaling pathways

**Methods:**

Male Wistar rats were divided into three main groups: sham, I/R+ Vehicle, and I/R+ Calcitriol. An experimental I/R model was created by occluding the middle cerebral artery (MCA) for 1 h, followed by a 72-h reperfusion period. Calcitriol (1 μg/kg) was administered intraperitoneally for three consecutive days post-stroke. Neurological deficit scores and infarct size were evaluated 72 h after MCAO. Gene expression levels of TLR4, MyD88, NF-κB, and FGFR2 in the brain cortex were measured using RT-PCR. Additionally, histopathological changes in the cortex were examined with Nissl staining. A molecular docking analysis was performed to investigate the interactions of calcitriol with TLR4 and FGFR2, providing insights into their binding affinities and potential functional implications.

**Results:**

Our findings indicated that calcitriol treatment significantly enhanced neurological function (P < 0.05) and reduced infarct volume (P < 0.001) in cerebral I/R injury. Furthermore, calcitriol decreased the number of damaged neurons while markedly increasing the count of neurons with normal morphology (P < 0.001). Consistent with the results from molecular docking showing that calcitriol antagonizes TLR4 and FGFR2, RT-PCR analysis also revealed that calcitriol significantly suppressed the upregulation of TLR4 (P < 0.05), MyD88 (P < 0.01), NF-κB (P < 0.01), and FGFR2 (P < 0.001) mRNA expression levels.

**Conclusion:**

The results demonstrate that calcitriol treatment offers significant neuroprotective benefits following cerebral I/R injury. These protective effects may be mediated, at least in part, by the inhibition of inflammation through the TLR4/MyD88/NF-κB and FGFR2 signaling pathways. This study enhances our understanding of the molecular mechanisms involved in calcitriol's neuroprotective actions.

## Graphical Abstract

**Figure:**
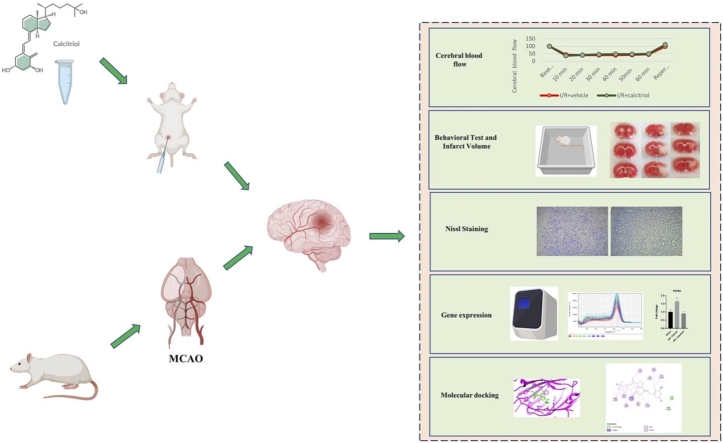

## 1 Introduction

Stroke is a leading cause of severe death and disability worldwide, characterized by high rates of morbidity, relapse, mortality, and impairment. It results from insufficient blood flow to the brain and is primarily categorized into two types: ischemic stroke and hemorrhagic stroke (Vahidinia et al., 2024). Notably, ischemic strokes account for 84.4 % of all stroke cases, as reported by the GBD 2016 Stroke Collaborators (2019) (Tadi and Lui, 2023). Currently, thrombolytic agents are the only clinically effective therapy for ischemic stroke; however, their application is limited by a narrow therapeutic window and safety concerns (Bonaventura et al., 2016). The underlying pathophysiological mechanisms of brain ischemic injury are intricate and not completely understood. Recent research has highlighted the critical role of inflammation in brain damage following ischemia (Bonaventura et al., 2016, Wu et al., 2017, Nejati et al., 2018).

Toll-like receptors (TLRs) are type I transmembrane proteins that are essential for triggering inflammatory responses by detecting damage-associated molecular patterns (DAMPs) and pattern-associated molecular patterns (PAMPs) (Iwasaki and Medzhitov, 2004, Kielian, 2006). Of the various Toll-like receptors, TLR4 has been the most extensively studied and plays a critical role in the pathogenesis of central nervous system (CNS) disorders (Feng et al., 2011). TLR4 engages with two specific adaptor proteins, myeloid differentiation primary response 88 (Myd88) and Toll-receptor-associated activator of interferon (TRIF), which activate parallel signaling pathways that start the transcription of factors involved in the regulation of pro-inflammatory cytokine gene expression (Buchanan et al., 2010). Furthermore, the TRIF-dependent pathway promotes late-phase activation of NF-κB, while the MyD88-mediated pathway triggers early activation of NF-κB. It has been demonstrated that neuroinflammatory responses mediated by the TLR4/MyD88/NF-κB pathway are crucial in cerebral ischemia/reperfusion (I/R) injury (Gao et al., 2009, Chen et al., 2018). Importantly, blocking the TLR4 signaling pathway has been proved to confer protection against ischemic stroke (Caso et al., 2007, Wang et al., 2014).

In recent years, the fibroblast growth factor receptor (FGFR) /fibroblast growth factors (FGFs) system has garnered significant attention in the study of various neurological diseases, including Alzheimer's disease, Parkinson disease, depression, multiple sclerosis (MS), epilepsy, anxiety, schizophrenia and others (Deng et al., 2019, Liu et al., 2021, Rajendran et al., 2021, Alam et al., 2022). The FGFR family includes four transmembrane tyrosine kinase receptors, known as FGFR1 to FGFR4, which interact with a group of FGFs. In the CNS of adult rats, FGFR1 and FGFR4 are primarily found in neurons, while FGFR2 and FGFR3 are more abundantly expressed in oligodendrocytes and astrocytes, respectively (Asai et al., 1993, Miyake et al., 1996). FGFR signaling is believed to have neuroprotective properties and to reduce neuroinflammation (Woodbury and Ikezu, 2014). Consequently, agonists of FGFR have been explored as potential therapeutic targets for conditions such as traumatic brain injury, Alzheimer's disease, Parkinson's disease, and others (Li et al., 2010). However, there are also reports of neurotoxic effects associated with FGFR signaling, including its role in inducing apoptosis in amyotrophic lateral sclerosis (ALS) (Cassina et al., 2005), and promoting axon degeneration in experimental autoimmune encephalitis (EAE) (Rajendran et al., 2021), highlighting its complex and varied functions across different neurological conditions. However, the expression patterns and functional roles of FGFRs in the context of ischemic stroke have been explored in only a limited number of studies.

Vitamin D as a new neuroactive steroid possesses neuroprotective and antioxidant properties, alongside its well-established roles in bone metabolism and the regulation of calcium-phosphate balance (Cui et al., 2019). It can be obtained either through food sources or synthesized in the skin from 7-dehydrocholesterol by ultraviolet light. Following two hydroxylation steps catalyzed by P450 enzymes, it is converted into its active form (calcitriol) (Jiang et al., 2014). Calcitriol exerts a wide range of physiological and pathophysiological effects by binding to the vitamin D receptor (VDR) (Cui et al., 2019). Evidence suggests that vitamin D plays a role in modulating various physiological functions, including immune system modulation, brain development and cell cycle control due to the VDR widespread presence (Eyles et al., 2013, Jorde et al., 2015). Additionally, vitamin D has been shown to reduce oxidative stress markers and inhibit the production of inflammatory cytokines in both in vitro and in vivo studies (Cui et al., 2019, Vahidinia et al., 2022). However, there is a limited understanding of the precise molecular mechanisms triggered by vitamin D treatment in the context of ischemic stroke.

This study aimed to explore the potential therapeutic effects of calcitriol in experimental rat models of brain ischemia, as well as to identify the possible mechanisms underlying these effects using both in vivo and in silico approaches.

## 2 Material and methods

### 2.1 Animals

The animal house of Kashan University of Medical Sciences provided male Wistar rats weighing 230–300 g. The animals were housed in plexiglass cages under controlled conditions, including a 12-h light/dark cycle, a temperature of 22 ± 2°C, and a humidity level of 50 ± 5 %, with free access to food and water. Kashan University of Medical Sciences approved ethical guidelines for conducting all experiments and animal care procedures under the ethics code IR.KAUMS.AEC.1402.019.

### 2.2 MCAO model and calcitriol treatment

To cause brain ischemia, the intraluminal filament method was used to perform middle cerebral artery occlusion (MCAO). The rats underwent anesthesia with 2 % isoflurane via an isoflurane vaporizer (Eickemeyer, Germany). Once the rats were entirely unconscious, they were placed supine on a heating pad (NARCO Bio-systems, USA) to maintain their body temperature at 37 ± 0.5°C and secured with adhesive tape. An incision was made in the middle of the neck, and the soft tissues were retracted. The right common carotid artery (CCA) dissection started from the rostral end. It proceeded until it split into the external carotid artery (ECA) and internal (ICA). After inserting a silicon-coated monofilament (Doccol, USA) into the CCA, it was advanced into the ICA until resistance was felt and the blood flow trace showed a marked reduction in CBF. At this point, the filament was employed to occlude the origin of the right middle cerebral artery (MCA). After one hour, the filament was carefully withdrawn to facilitate reperfusion of the MCA. The success of the occlusion was verified by monitoring cerebral blood flow (CBF) using laser Doppler flowmetry (Moor Instruments VMS-LDF2, UK) in the ipsilateral cortex. The probe tip was secured with an adhesive on the intact skull above the ischemic cortex, positioned 2 mm posterior and 5 mm lateral to the bregma. In the end, all the incisions were closed, and the animals were given time to recover from their anesthesia before being returned to their cage during the reperfusion period. Animals that did not survive the procedure or failed to exhibit at least a 50 % reduction in cerebral blood flow (CBF) following ischemia induction were excluded from the study.

Three groups of animals were randomly selected, including A) the sham-operated (sham) group (n = 6), B) the vehicle-treated tMCAO (I/R+vehicle) (n = 6), and C) calcitriol-treated tMCAO (I/R+calcitriol) (n = 6). The rats in the sham group received the same surgical procedure as the others, but they did not have MCA occlusion. The rats were administered an intraperitoneal injection of calcitriol (Cayman Chemical Company, USA) that was initially dissolved in ethanol at a concentration of 1 mg/ml and then immediately diluted with saline before injection. This treatment was given at a dosage of 1 µg/kg body weight for three consecutive days following stroke induction (administered at 30 min, 24 h, and 48 h post-ischemia) (Cui et al., 2017). The vehicle group received an equivalent volume of saline mixed with the required amount of ethanol at the same time. Behavioral tests were conducted 72 h after MCAO modeling. Subsequently, the rats were euthanized by decapitation, and their brains were extracted for further analyses.

### 2.3 Behavioral measurements

Neurological deficits were evaluated using the Garcia score, an 18-point assessment scale commonly employed to quantify functional impairments before and after MCAO [28]. Six behavioral aspects are evaluated in this neurological examination, including spontaneous activity, walking, climbing, fore-paw outstretching, body proprioception, and reaction to vibrissae touch. The total score was calculated by summarizing the scores after conducting the tests (Vahidinia et al., 2021). A lower score was a sign of more severe dysfunction. All sham-operated animals scored 18 points, indicating no functional deficits

### 2.4 Measurements of infarct volume

2,3,5-Triphenyltetrazolium chloride (TTC) staining (Merck, Germany) was performed according to previously established protocols. Brains were rapidly extracted 72 h after MCAO and frozen for 30 min. Five coronal sections with a thickness of 2 mm were incubated in 1 % TTC solution prepared in PBS for 20 min at 37 °C. Ultimately, the brain slices were photographed with a digital camera (SONY, Japan). The volume of each slice's corrected cerebral infarction was determined using the equation below, and swelling corrections were made: %Infarction volume= (contralateral hemisphere volume−ipsilateral hemisphere non-injured volume)× 100/contralateral hemisphere volume.

### 2.5 Quantitative real-time PCR

Seventy-two hours post-MCAO, the rats were sacrificed, and the ischemic cortical tissue was carefully harvested. Total RNA was extracted using TRIzol reagent (MaxZol, Iran) following the manufacturer's protocols. The isolated RNA was then reverse transcribed into complementary DNA (cDNA) using cDNA Synthesis Kit (Parstous, Iran). Quantitative real-time PCR (RTqPCR) was performed using SYBR Green qPCR Master Mix with high ROX (Ampliqon, Denmark) and detected by an Applied Biosystems Real-Time PCR System (Thermo Fisher Scientific, USA). HPRT was used as a house-keeping gene to determine the cDNA levels. The relative quantities of mRNA were normalized to HPRT (internal control) using the 2^-△△Ct^ method. All the primers used were listed in Table 1.

**Table 1:** Sequences of PCR primers used for Real-Time PCR.

**Primer name**	**Sequence (5′ to 3′)**	**Size (bp)**
**TLR4**	Forward: CCTCGAGTGGGAGGACAATG Reverse: TCCCTGTCCACAGCAGAAAC	219
**Myd 88**	Forward: AAATTGTGTGTGTCCGACCG Reverse: GGATCAGTCGCTTCTGTTGG	193
**NF–kB**	Forward: TGACGGGAGGGGAAGAAATC Reverse: AAACACGGAAGCTGGCTTTG	206
**FGFR2**	Forward: GACAGCACCAGGAACCTACT Reverse: CTTGCGGCTGTCCACTTATC	199
**HPRT**	Forward: GCTCGAGATGTCATGAAGGAGA Reverse: TCAGCGCTTTAATGTAATCCAGC	109

### 2.6 Tissue preparation

Upon completion of behavioral tests, the rats were sacrificed for histological analysis. They were first anesthetized with 10 % chloral hydrate and followed by transcardial perfusion with 0.9 % normal saline, then with 10 % neutral buffered formalin (10 % NBF). Brains were extracted and post-fixed in 10 % NBF at 4 °C for 48 h. Afterward, the brain samples were embedded in paraffin, and coronal sections were cut to a thickness of 5 μm before being stained with Nissl for examination under a light microscope (Nikon Eclipse Ti-U, Japan)

### 2.7 Nissl staining

Nissl staining was utilized to analyze morphological changes in neurons in the ischemic penumbra 72 h after reperfusion. The tissue sections were first deparaffinized and then washed with varying concentrations of ethanol followed by distilled water. Next, the slices were stained for 10 min at 37°C in a preheated solution of 0.05 % (w/v) cresol violet. After staining, the sections were quickly rinsed with distilled water and differentiated in a 95 % ethanol solution for 1 min. Subsequently, the sections were dehydrated in 100 % ethanol, cleared with xylene, and mounted with neutral gum. The stained slices were photographed. Then, the total number of intact neurons in the penumbra was counted in three different fields of view for each section by an observer blinded to the groups using light microscopy (Nikon Eclipse Ti-U, Japan).

### 2.8 Molecular docking

Molecular docking is a common technique for investigating drug-protein interactions. In this study, AutoDock Vina was employed to evaluate the binding affinity of calcitriol with two critical protein targets: the TLR4/MD-2 complex and FGFR2. Crystal structures of the TLR4-MD2 complex (PDB code, 3FXI) and FGFR2 (PDB code, 1E0O) were obtained from the RCSB Protein Data Bank (http://www.rcsb.org/). The proteins and ligands were processed using Chimera and AutoDockTools. The binding energy was calculated using AutoDock Vina.

### 2.9 Statistical analysis

All values are presented as mean±SD. Analysis of variance (ANOVA) was used to compare data between groups, followed by a Turkey post-hoc. P < 0.05 was considered as a significant difference for all the comparisons.

## 3 Results

### 3.1 Regional cerebral blood flow (rCBF) monitoring during the tMCAO procedure

**Fig. 1:**
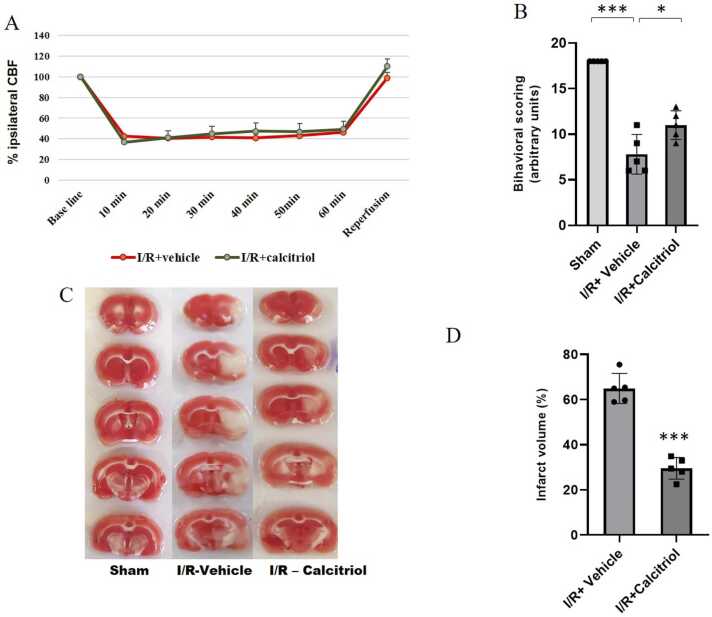
CBF course, behavioral scores and infarction volume. CBF was recorded with a laser Doppler flowmeter before and during MCAO. (B) Neurobehavioral function was assessed using the Garcia test, showing significant improvement in calcitriol-treated rats with transient MCAO compared to vehicle-treated rats (*P* < 0.001 *versus* sham group and *P* < 0.05 *versus* vehicle group). (C and D) Representative images of TTC-stained brain sections and corresponding quantitative measurements of cerebral infarction volumes. No ischemic lesions were observed in the sham rats. In the vehicle-treated group, TTC-stained brain slices revealed a significant infarct region, while calcitriol treatment notably reduced the infarct volume. Data are presented as mean ± SD (*P* < 0.001 *versus* vehicle group; n = 5 in each *group*).

Representative changes in regional cerebral blood flow (rCBF) as a percentage of baseline over time during pre-ischemia, ischemia, and reperfusion are depicted in Fig. 1A. Baseline blood flow was assessed using Laser-Doppler monitoring prior to tMCAO, establishing a baseline of 100 % for all animals. During the procedure, the sham-operated group exhibited no significant changes in CBF at any time point. In contrast, in the I/R rats receiving either vehicle or calcitriol, CBF decreased to below 50 % of baseline immediately after occlusion and remained stable throughout the ischemic phase. Following removal of the monofilament, blood flow returned to over 90 % of baseline across all I/R groups, with no significant differences detected among them.

### 3.2 Calcitriol mitigates neurological deficits and infarct size in rats subjected to stroke

To evaluate the neuroprotective effects of calcitriol on ischemic injury, neurological deficits were assessed 72 h post-reperfusion, as shown in Fig. 1B. The sham group exhibited no significant neurological deficits. However, the vehicle-treated group showed a marked increase in neurological deficits (P < 0.001) compared to the sham group. Importantly, calcitriol-treated rats displayed a significant drop in behavioral dysfunction compared to those in the vehicle group (11 ± 1.6 vs 7.8 ± 2.2; P < 0.05).

Cerebral infarction was evaluated through TTC staining 72 h after reperfusion. In Fig. 1C, the infarcted areas following I/R are depicted in white, while regions of the cortex with viable tissue appear red. The volume of the infarct area was quantified using ImageJ software. No infarction was observed in the rat brains of the sham group. In comparison to the sham group, the infarct volume percentage was significantly higher in the vehicle group (Fig. 1D). The vehicle group exhibited an infarct volume percentage of 64 % and treatment with calcitriol reduced the infarct volume significantly to 30 % compared to the vehicle group (29.6 ± 4.8 vs 64.91 ± 6.6; P < 0.001).

### 3.3 Calcitriol alleviates neuronal morphologic damage in stroke rats

**Fig. 2:**
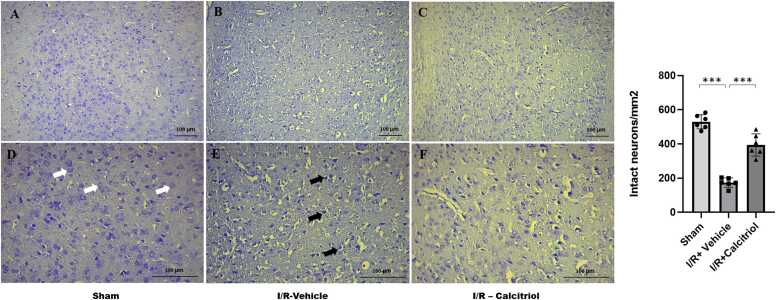
Representative Cresyl Violet-stained images of brain tissue sections from different experimental groups: (A) Sham, (B) I/R + vehicle, and (C) I/R + calcitriol. (A, D) The Sham group displayed normal neurons (white arrow). (B, E) Rats subjected to MCAO and treated with the vehicle exhibited frequent shrinkage and pyknotic (dead) cells (black arrow). (C, F) The I/R + calcitriol group showed a reduced number of dead neurons (black arrow). Data are presented as mean±SD (*P* < 0.001 *versus* sham group and *P* < 0.001 *versus* vehicle group; n = 6 in each *group*).

Nissl staining was used to observe the damage to the neurons. Fig. 2 displays the Nissl staining results. In the cortex of rats that underwent sham operations, the neurons exhibited normal morphology and structure, with abundant Nissl material. In contrast, neurons in the cortex of rats subjected to MCAO displayed significant atrophy; their nuclei were deeply stained, and the cells had shrunken bodies along with pyknotic nuclei. Conversely, the calcitriol-treated group exhibited a significant decrease in damaged neurons and a notable increase in neurons with normalized morphology (394.3 ± 65.04 vs 173.5 ± 27.4; P < 0.001).

### 3.4 The calcitriol effects on the mRNA levels of TLR4, MyD88, NF-kB and FGFR2 in the MCAO rat brain tissue

**Fig. 3:**
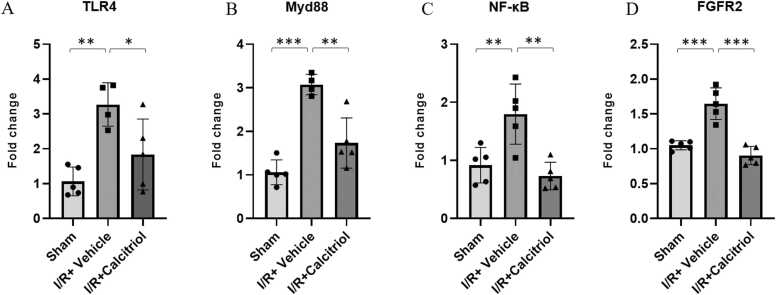
The impacts of calcitriol on TLR4, MyD88, NF-κB and FGFR2 mRNA expression. (A) The TLR4, MyD88, NF-κB and FGFR2 expression levels were upregulated in vehicle group and significantly reduced after treatment with calcitriol when comparing with vehicle group Data are presented as mean±SD (*P* < 0.01 and *P* < 0.001 *versus* sham group and *P* < 0.05, *P* < 0.01 and *P*^<^0.001 *versus* vehicle group; n = 5 in each *group*).

To investigate how calcitriol mediates its therapeutic effects against ischemic injury, we analyzed the involvement of the TLR4/MyD88/NF-κB and FGFR pathways by measuring the expression levels of these genes using real-time PCR (Fig. 3). The MCAO group showed a substantial increase in the mRNA levels of TLR4 (3.3 ± 0.6 vs 1.07 ± 0.4; P < 0.01), MyD88 (3.07 ± 0.24 vs 1.06 ± 0.3; P < 0.001), NF-κB (1.8 ± 0.52 vs 0.9 ± 0.3; P < 0.01), and FGFR2 (1.7 ± 0.23 vs 1.05 ± 0.07; P < 0.001) compared to the sham group. Conversely, the calcitriol-treated groups exhibited a noticeable decrease in the mRNA levels of these genes compared to the MCAO group (TLR4 (1.84 ± 1.02 vs 3.3 ± 0.6; P < 0.05), MyD88 (1.73 ± 0.6 vs 3.07 ± 0.24; P < 0.01), NF-κB (0.73 ± 0.24 vs 1.8 ± 0.52; P < 0.01, and FGFR2 (0.9 ± 0.13 vs 1.7 ± 0.23; P < 0.001).

### 3.5 Molecular docking analysis of calcitriol with TLR4 and FGFR2

**Fig. 4:**
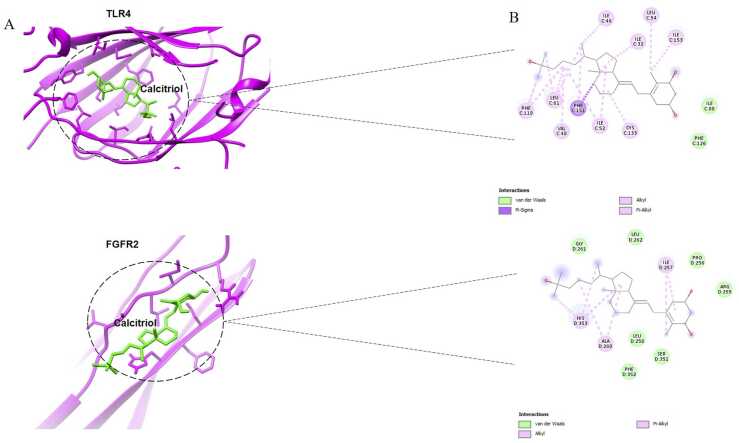
Molecular docking interactions between TLR4, FGFR2, and calcitriol. (A) 3D representation of the interactions between TLR4, FGFR2, and calcitriol. (B) 2D representation of the interactions between TLR4, FGFR2, and calcitriol.

Molecular docking was performed to predict the potential therapeutic effects of calcitriol by evaluating its binding energy with key protein targets. Molecular docking by AutoDock Vina demonstrated that calcitriol was in the pocket of the TLR4-MD2 complex and FGFR2 protein (Fig. 4). Lower binding energies typically indicate strong interactions and high affinity between the ligand and receptor. The binding energies between calcitriol and TLR4-MD2 complex and FGFR2 protein were −8.6 kcal/mol, and −5.1 kcal/mol, respectively. These results suggest that calcitriol may exhibit inhibitory activity against inflammation through its interactions with these targets. In general, affinity values greater than −5 kcal/mol indicate a lack of predicted binding, values between −5 kcal/mol and −7 kcal/mol suggest moderate predicted binding, while values below −7 kcal/mol imply strong predicted binding (Pantsar and Poso, 2018). These findings suggest that calcitriol suppresses the TLR4 and FGFR2 signaling pathway, and these receptors may be a potential target of calcitriol by directly binding with calcitriol

## 4 Discussion

The present study showed that calcitriol reduced ischemic complications through the inhibition of TLR4 signaling via the MyD88-dependent pathway and the FGFR2 Pathway to protect against cerebral I/R injury. Ischemic stroke represents a significant threat to global health (Bowren Jr et al., 2022). Intravascular thrombectomy and intravenous thrombolysis are the main methods for conserving penumbra cells and decreasing infarct volume after an ischemic stroke (Herpich and Rincon, 2020). Restoring blood flow can exacerbate brain damage, leading to the risk of hemorrhage and cerebral edema, which is known as I/R injury (Yang et al., 2021). There is an urgent need for innovative treatment strategies to mitigate reperfusion injury in patients with ischemic stroke. Therefore, investigating neuroprotective agents with diverse mechanisms may offer significant benefits for those suffering from acute ischemic stroke.

In several studies, neurosteroids have been shown to be neuroprotective agents against cellular damage caused by ischemia (Tajalli-Nezhad et al., 2019, Xu et al., 2022). Calcitriol, a fat-soluble steroid hormone, is the active form of vitamin D in the body that binds to VDRs (Cui et al., 2017). Neurological and cerebrovascular diseases are linked to vitamin D deficiency (Daubail et al., 2014), and evidence suggests that it has neuroprotective effects in ischemic stroke (Han et al., 2015). Studies reveal that patients with acute stroke often exhibit vitamin D deficiency, which correlates with larger brain infarction volumes and more severe behavioral disturbances (Balden et al., 2012). Since stroke is the primary cause of death in patients with vitamin D deficiency, long-term vitamin D therapy may have the potential to decrease both stroke risk and mortality rates (Pilz et al., 2011, Daubail et al., 2014). Numerous studies have identified various mechanisms through which calcitriol offers protection, including anti-inflammatory and anti-apoptotic properties, vasodilation, and the reduction of arterial pressure (Kajta et al., 2009, Guo et al., 2019, Khassafi et al., 2022), the precise neuroprotective mechanisms of calcitriol following ischemic stroke are still not well understood. There is substantial evidence suggesting that calcitriol decreases the size of infarctions in MCAO model rats (Fu et al., 2013, Sadeghian et al., 2019, Qiao et al., 2023). The effectiveness of a treatment can be determined by performing this first step, which was also carried out in this study. Our evaluation of infarct volumes and neurological defects after ischemia revealed that calcitriol therapy could be advantageous as a modulator of ischemia. Following previous reports, our study found that calcitriol treatment significantly decreased the volume of brain lesions while improved functional deficits (Fig. 1). It has been shown that acute administration of calcitriol—given 4 h after stroke induced by endothelin-1 and then every 24 h for 5 consecutive days— failed to improve cerebral damage or neurobehavioral functions [36]. The discrepancies between reported results may be attributed to differences in the timing of vitamin D administration or the use of varying experimental models (Balden et al., 2012).

Inflammation plays a significant role in secondary brain injury following ischemic stroke, making it a potential therapeutic target (Anrather and Iadecola, 2016). Among the various TLRs, TLR4 has been extensively studied and is notably activated after cerebral ischemia. Inhibiting TLR4 has been shown to effectively reduce inflammation after an ischemic stroke (Andresen et al., 2016). TLR4 activation results in the recruitment of MyD88, which enables NF-κB to move from the cytoplasm to the nucleus and initiate inflammatory responses (Hua et al., 2009). This TLR4/MyD88/NF-κB-mediated inflammatory pathway is critical in causing neuronal damage during ischemia/reperfusion (I/R) (Tao et al., 2016).

Beyond its well-known role in calcium metabolism, vitamin D possesses powerful immunomodulatory effects that can influence the immune response to injury in various diseases (Schedel et al., 2016, Qiao et al., 2023). Previous studies have explored the relationship between calcitriol and TLR4 in vitro and in vivo experiments. It has been found in these investigations that calcitriol can influence TLR signaling pathways and inhibit NF-κB activity, resulting in the suppression of pro-inflammatory factors (Du et al., 2009, Chen et al., 2013, Jeong et al., 2014). A study on calcitriol-treated monocytes demonstrated that vitamin D influences TLRs by inhibiting the nuclear translocation of NF-kB/RelA and suppressing p38 and p42/44 (ERK1/2) stress-activated protein kinase (SAPK) signaling in purified monocytes when TLR ligands are engaged (Sadeghi et al., 2006). Min Sung Kim and coworkers found that paricalcitol, a vitamin D analog, reduces hepatic I/R injury by downregulating the TLR4/NF-κB signaling pathway in rats (Kim et al., 2017). Tajalli-Nezhad et al. found that a single dose of calcitriol administered 24 h after a stroke significantly reduces the expression of TLR4/2 and NF-κB, which leads to decreasing the levels of inflammatory factors in the ischemic stroke patients (Tajalli-Nezhad et al., 2023). In this study, we utilized molecular docking to investigate whether calcitriol could serve as a targeted drug against TLR4 and potentially improve cerebral I/R injury by modulating inflammatory pathway. The docking analysis revealed that the binding energies between calcitriol and TLR4 were consistently below −8 kcal/mol, indicating strong binding affinity. Structural analysis further demonstrated that calcitriol interacts with the binding sites of TLR4/MD-2 through hydrophobic interactions. In consistent with docking results, real-time experiments showed that calcitriol could modulate TLR4/MyD88/NF-κB signaling pathway. Using computational methods (http://jaspar.genereg.net/), it has been shown that TLRs have multiple vitamin D -response elements (VDRE) in their gene sequence, suggesting a possible direct regulation of vitamin D in the expression of TLRs. Thus, calcitriol may have a dual role; in addition to downregulating gene expression, it could also inhibit the activity of the TLR4 receptor (Martínez-Moreno et al., 2020). Thus, calcitriol may have a dual role; in addition to downregulating gene expression, it could also inhibit the activity of the TLR4 receptor.

Fibroblast growth factors (FGFs) and their high-affinity tyrosine kinase receptors (FGFR1-FGFR4) play essential roles in tissue and organ homeostasis, repair, and development. Abnormal expression or activity of these receptors has been associated with a wide range of diseases and disorders (Hui et al., 2018). Following activation, FGFRs trigger both canonical and non-canonical signaling pathways. Canonical pathways involve the activation of ERK and PI3K through FRS2α, as well as FRS2α-independent activation of PLC-γ (Wang et al., 2016, Wang et al., 2019). On the other hand, non-canonical pathways, which are less extensively studied, include post-translational modifications of LDHA and TAK1, enhancing the activity and stability of these molecules (Liu et al., 2018, Wang et al., 2018). Given the broad functionality of FGFRs, developing compounds that specifically target these receptors has become a major area of research.

Research has shown that FGFR inhibition can reduce inflammation and mitigate liver damage by suppressing the TNFα–NF-κB signaling pathways (Wang et al., 2020). Conversely, another study revealed that calcitriol affects both the expression and phosphorylation of FGFR receptors. In metastatic RPMI7951 melanoma cells, calcitriol treatment led to an upregulation of FGFR2 mRNA levels (Piotrowska et al., 2024). Research has demonstrated that endogenous FGFR1 and FGFR2 offer intrinsic protection to cardiomyocytes during I/R injury by minimizing cell death and suppressing hypertrophy, ultimately improving functional outcomes. The lack of FGFR1 and FGFR2 in cardiomyocytes worsens the impact of I/R injury, resulting in heightened myocyte death within 24 h and reduced cardiac function after a week (Matsiukevich et al., 2022). Yan et al. revealed that a dietary deficiency of vitamin D3 decreases the mRNA expression of FGFRs (FGFR1–4) and VDR in the calvaria (Yan et al., 2022). In recent years, strategies aimed at modulating the FGF/FGFR pathway have achieved moderate success in reducing symptoms in animal studies. Particularly, the targeted removal of FGFR1&2 has demonstrated considerable therapeutic promise (Mierzwa et al., 2013, Kamali et al., 2021). The targeted deletion of FGFR2 in oligodendrocytes specifically led to improvements in motor function, reduced myelin and axonal damage, and lower inflammation in a mouse model of MS. These positive outcomes are attributed to the modulation of downstream proteins within the FGF/FGFR signaling pathway, including pERK and pAkt, which play key roles in myelination and inflammatory processes (Kamali et al., 2021). The study showed that the FGFR-specific inhibitors AZD4547 and dovitinib successfully inhibited the FGFR signaling pathway in oligodendrocytes in vitro. This inhibition enhanced BDNF/TrkB signaling and promoted remyelination by boosting the production of myelin-specific proteins. As a result, FGFR inactivation appears to be a promising protective approach for slowing the progression of MS (Rajendran et al., 2023). Despite the abundance of research on the effects of FGFRs in various diseases, there is no report of FGFR2 role in ischemic stroke. We observed for the first time that the expression of FGFR2 gene was higher in ischemia-induced cortical penumbra compared to the sham group, while its expression was reduced in the calcitriol-treated group. We also evaluated the interaction between calcitriol and FGFR2 in the present study by molecular docking. Analysis of the docking results revealed that calcitriol effectively docked into the FGFR2 binding site, exhibiting significant interactions with its critical amino acid residues. Overall, our findings demonstrate that calcitriol directly binds to FGFR2, suppressing its elevated expression levels induced by ischemia. This interaction further attenuates NF-kB pathway activation and reduces the expression of pro-inflammatory cytokines.

Although no studies have specifically investigated the relationship between FGFR2 and TLR4, research has demonstrated that high glucose -induced activation of FGFR1 is mediated by TLR4 and c-Src in the context of cardiomyopathy. This signaling pathway ultimately leads to MAPK-mediated activation of NF-κB, resulting in increased transcription of inflammatory cytokines, which contributes to fibrosis and hypertrophy. Notably, both cardiomyocyte-specific FGFR1 knockout and pharmacological inhibition of FGFR1 have demonstrated significant protective effects against diabetes-induced cardiac inflammation (Chen et al., 2024). Given that FGFR1 has the closest homology with FGFR2 (Powers et al., 2000, Ferrarese et al., 2024), it is plausible that FGFR2 may interact with TLR4 in a manner similar to FGFR1.

### 4.1 Limitations

This study has limitations that should be acknowledged. First, while we demonstrated significant changes in mRNA expression of TLR4, MyD88, NF-κB, and FGFR2, protein-level validation using Western blotting or immunohistochemistry was not performed. As mRNA expression does not always correlate directly with protein abundance or functional activity, future studies must confirm these findings at the proteomic and post-translational levels. Second, although molecular docking suggested a strong binding affinity of calcitriol to TLR4 and a moderate affinity to FGFR2, this in silico approach does not account for receptor dynamics or competitive binding under physiological or ischemic conditions. Additional studies, such as ligand competition assays, are warranted to validate these interactions.

## 5 Conclusion

In conclusion, molecular docking combined with in vivo evaluation showed that the calcitriol administration can enhance neurological function, reduces infarct size, and alleviates cortical damage in rats following ischemic stroke. Additionally, calcitriol therapy helps mitigate the inflammatory response after I/R by inhibiting the TLR4/MyD88/NF-κB and FGFR2 pathways. This study confirms the therapeutic potential of calcitriol for ischemic stroke and explores its underlying mechanisms, presenting a promising treatment option for cerebral ischemic injuries.

## CRediT authorship contribution statement

**Hassan Hassani Bafrani:** Writing – review & editing, Conceptualization, Supervision. **Zeinab Vahidinia:** Supervision, Project administration, Writing – review & editing, Writing – original draft. **Sayyed Alireza Talaei Zavareh:** Writing – review & editing, Methodology, Formal analysis. **Reza Bayat:** Writing – review & editing, Investigation, Software. **Javad Amini Mahabadi:** Writing – review & editing, Methodology, Investigation. **Fahimeh Ramshini:** Investigation, Writing – original draft.

## Consent to participate

Not applicable

## Consent to publish

Not applicable

## Ethical statement

All of the experimental procedures were approved by the Ethical Committee for Research at Kashan University of Medical Sciences (IR.KAUMS.AEC.1402.019).

## Funding

This work was supported by a grant (Grant NO. 402169) from 10.13039/501100004048Kashan University of Medical Sciences.

## Conflicts of Interest

The authors have no relevant financial or non-financial interests to disclose.

## Data Availability

The data that support the findings of this study are available from the corresponding author upon reasonable request.
